# Knowledge-to-action processes in SHRTN collaborative communities of practice: A study protocol

**DOI:** 10.1186/1748-5908-6-12

**Published:** 2011-02-11

**Authors:** James Conklin, Anita Kothari, Paul Stolee, Larry Chambers, Dorothy Forbes, Ken Le Clair

**Affiliations:** 1 Department of Applied Human Sciences, Concordia University, Montreal, Quebec, Canada; 2 Élisabeth Bruyère Research Institute, Ottawa, Canada; 3 Department of Health Sciences, University of Western Ontario, London, Ontario, Canada; 4 Department of Health Studies and Gerontology, Faculty of Applied Health Sciences, University of Waterloo, Waterloo, Ontario, Canada; 5 Department of Epidemiology and Community Medicine, University of Ottawa, Ottawa, Ontario Canada; 6 Faculty of Nursing, University of Alberta, Edmonton, Alberta, Canada; 7 Division of Geriatric Psychiatry, Department of Psychiatry, Queens University, Kingston, Ontario, Canada

## Abstract

**Background:**

The Seniors Health Research Transfer Network (SHRTN) Collaborative is a network of networks that work together to improve the health and health care of Ontario seniors. The collaborative facilitates knowledge exchange through a library service, knowledge brokers (KBs), local implementation teams, collaborative technology, and, most importantly, Communities of Practice (CoPs) whose members work together to identify innovations, translate evidence, and help implement changes.

This project aims to increase our understanding of knowledge-to-action (KTA) processes mobilized through SHRTN CoPs that are working to improve the health of Ontario seniors. For this research, KTA refers to the movement of research and experience-based knowledge between social contexts, and the use of that knowledge to improve practice. We will examine the KTA processes themselves, as well as the role of human agents within those processes. The conceptual framework we have adopted to inform our research is the Promoting Action on Research Implementation in Health Services (PARIHS) framework.

**Methods/design:**

This study will use a multiple case study design (minimum of nine cases over three years) to investigate how SHRTN CoPs work and pursue knowledge exchange in different situations. Each case will yield a unique narrative, framed around the three PARIHS dimensions: evidence, context, and facilitation. Together, the cases will shed light on how SHRTN CoPs approach their knowledge exchange initiatives, and how they respond to challenges and achieve their objectives. Data will be collected using interviews, document analysis, and ethnographic observation.

**Discussion:**

This research will generate new knowledge about the defining characteristics of CoPs operating in the health system, on leadership roles in CoPs, and on the nature of interaction processes, relationships, and knowledge exchange mechanisms. Our work will yield a better understanding of the factors that contribute to the success or failure of KTA initiatives, and create a better understanding of how local caregiving contexts interact with specific initiatives. Our participatory design will allow stakeholders to influence the practical usefulness of our findings and contribute to improved health services delivery for seniors.

## Background

Across Canada, health planners are preparing for significant new numbers of seniors. Today seniors account for 13.7% of our population; by 2035 this will increase by approximately 25% [1]. Life expectancy is estimated at 83.2 years for men and 86.4 years for women [2]. Toward the end of life, many seniors experience a variety of disabilities and chronic diseases, including arthritis, high blood pressure, dementia, and incontinence [1]. About 35% of Canadians over 85 are living with dementia [1], a disease with major implications for the health system and informal caregivers [3].

As baby boomers retire, Ontario and other Canadian health jurisdictions are focusing on improving services and building capacity in aging and health. One way to do this is to improve the system's ability to generate, share, and use knowledge and innovations.

### Seniors health research transfer network (SHRTN) collaborative

Since its launch in 2005, the SHRTN Collaborative has become a significant knowledge network linking Ontario caregivers, policy makers and researchers who focus on improving the care of seniors. The SHRTN Collaborative is a network of networks that includes the SHRTN Knowledge Exchange, Alzheimer Knowledge Exchange, and Ontario Research Coalition [4]. These networks facilitate knowledge exchange through a library service, knowledge brokers (KBs), local implementation teams, collaborative technology, and Communities of Practice (CoPs). The more than 8,500 CoP members identify innovations, translate evidence, and implement changes in health settings to improve seniors' health [5].

SHRTN carries out an evaluation process to promote the development and strengthening of the network and its components [6]. This evaluation has helped network leaders to develop a relatively stable organizational structure with specific components and activities contributing to the network's success. Now that the network has achieved this stability, we have developed this research program to better understand and enhance the network's Knowledge-to-Action (KTA) processes.

### Exchange approaches to KTA

Health outcomes tend to improve if research is used consistently and appropriately in caregiving organizations [7-10]. This has led to more research focusing on how scientific and practice-based knowledge move into frontline practices. We thus use the term KTA [7] because it leaves open the source of the knowledge (in scientific inquiry or field experience) and the identity of the knowledge user (patients, family members, policy makers, caregivers, educators, *et al.*).

Many researchers argue that knowledge adoption involves interaction and engagement, and is more iterative than linear [11-15]. Some see the movement of knowledge into practice as involving the systematic interaction of several key elements, including the people who are considering adopting the new knowledge, the practice contexts where these people work, the characteristics of the knowledge that is being adopted, and the strategies used to facilitate adoption [16]. Others call for collaboration between researchers and practitioners to improve knowledge dissemination [13,17-21].

Research has also shown that KTA processes can involve clashing priorities and values [22], and are influenced by factors within local contexts [23]. Some studies suggest that KTA is impacted by the unique characteristics of the stakeholders, evidence, and organizations participating in the exchange [24,25]. McWilliam and colleagues suggest that social interaction takes various forms during KTA implementation [26]. Some suggest that KTA is a process of negotiating between knowledge derived from different sources [27-30]. Estabrooks and colleagues argue that explicating KTA processes requires a variety of theoretical lenses [31].

A similar conception of KTA is found in the Promoting Action on Research Implementation in Health Services (PARIHS) theory, which sees KTA as dependent upon the interplay between three factors: the level and nature of the evidence being transferred, the organizational context that is implementing the evidence, and the method of facilitating the implementation process [32-38].

Greenhalgh and colleagues concluded that adopting new knowledge involves an interaction between knowledge, individual adopters, and organizations where the adoption occurs [39]. They call for more research on specific local settings to reveal factors that influence the implementation of innovations, and for research on how a local context interacts with a knowledge transfer program. This research should be reported in detailed descriptive reports to present the unique features of the local contexts being studied, using participatory designs so members of the local context can influence the practical usefulness of the findings.

PARIHS researchers call for 'communities of researchers, practitioners, and other stakeholders undertaking pieces of work to test the whole [PARIHS] framework as presented as a way of moving the agenda forward. We see the need for this collaborative approach, not only between researchers but also between research teams and those practitioners at the local level who actually have the task of implementing evidence into practice' [34]. Our proposal answers this call, and meets the need identified by Greenalgh and colleagues to catalogue and potentially enhance KTA processes as they enter specific healthcare organizations [39].

### CoPs as Mobilizers of KTA

At the same time that many researchers have come to favour an interaction theory of knowledge translation, and to focus on the role of factors such as organizational context and facilitation processes, others have been looking at specific organizational forms that appear to promote knowledge translation. One such form is the CoP.

The notion of CoPs is based on a view of learning as an individual and social phenomenon. Early theorists of social learning suggested that learning is not a matter of transferring knowledge from experts to novices, but is rather a complex process embedded in social interaction [40,41]. These views are evident in Kolb's learning cycle which depicts four phases of learning through experience, and Taylor's model which posits a transitional process provoked by moments of disorientation [42,43]. Schön's concept of the reflective practitioner sees learning as involving ongoing interactions between practitioners as they work to solve the daily problems of practice [44]. Extending these insights, some researchers have examined social learning *in situ*, with attention focused on knowledge sharing in CoPs. These researchers often argue that learning is a characteristic process within a practice that creates the community's adaptability and stability [45-51], and fosters the creation, use, and retention of knowledge (often in the form of tools and shared narratives) conceived of as collective property [49,52,53]. This view of learning has been opposed to a view of learning as involving a one-way transfer of formal knowledge between groups or individuals [45,54-57].

Learning in a CoP involves participation, which speaks to the experience of belonging to a practice, and includes accomplishing tasks while interacting with colleagues. It also involves reification, which speaks to the tools of the practice (techniques and documents, *et al.*, that are used while doing the work). Some argue that a CoP experiences an ongoing dynamic between stability and adaptation [50,51,53,58,59]. The practice creates tools to maintain its competence and make it easier to do its work [51,60,61]. Simultaneously, the practice adapts to change through interaction between insiders and outsiders, and through the turnover of members [48,51]. The result is the collective knowledge of the community that is both contextual and local [45,62-64]. It is largely tacit, and passes among members through ongoing interaction [61,65-69]. It derives chiefly from experiences, is expressed through experimentation, and is often sustained through narratives of past challenges and solutions [49].

Some have noted, however, that although policy makers and practitioners are adopting CoPs as a vehicle for moving new knowledge into practice, the concept of CoPs, and the precise way in which these communities mobilize KTA processes, is not fully understood [70-73]. Li and colleagues call for research to shed light on the precise characteristics of new and mature CoPs, and for a focus on optimizing community attributes such as interaction processes, relationship building, and knowledge exchange in ways that promote higher levels of performance [71,72].

Much of the work on CoPs has focused on how a community creates new knowledge to solve the challenges of its shared enterprise. In the case of the SHRTN Collaborative, CoPs mobilize knowledge that is then moved toward frontline practices, where it is hoped that the knowledge will be implemented. This model resembles that of Wenger and colleagues, where the interplay between action in practice is balanced by reflective learning among members of a CoP who may belong to different practices [74]. There are, however, two differences between this conceptualization and the CoPs operating within the SHRTN Collaborative. First, SHRTN CoPs are not simply a context for reflective practice, but also often explicitly seek to link a frontline practice with relevant research evidence. Second, SHRTN CoPs operate within the context of a knowledge network, and may benefit from some of the cohesive mechanisms that have evolved to allow network participants to learn about and adapt to best practices in knowledge exchange. To date, little research has been done to describe how KTA processes unfold through CoPs that exist outside of, but adjacent to, the frontline setting, and how operating within a network framework might impact upon CoP performance.

The SHRTN Collaborative defines a CoP as 'a group of people who come together to exchange information and knowledge on a specific topic related to seniors' health and health care' [5]. CoP members include caregivers, policy makers, researchers, educators, librarians and others. Each CoP has a core group of leaders and a larger group of members who participate in CoP activities, with leaders and members located in different organizations throughout Ontario. CoP leaders mobilize relevant knowledge to solve the compelling problems of frontline practice. The CoPs have access to a KB (who helps to assemble relevant knowledge, and facilitate the implementation of the knowledge), a library service, and online collaboration tools. To move knowledge into action, CoPs have used numerous facilitative techniques, including: forming collaboratives that share experiences and experiment with solutions; holding webinars on special topics; hosting regional conferences to share ideas and form partnerships; and responding to requests to identify evidence that might be used to solve specific problems.

In 2008 and 2009, the SHRTN Collaborative provided funding support to 19 CoPs on topics such as communicative access and aphasia, activity and aging, continence care, elder abuse, aging and developmental disabilities, and end of life care.

### Research objectives

This project aims to increase our understanding of the KTA processes mobilized through CoPs that are working to improve the health of Ontario seniors. KTA refers to the movement of research and experience-based knowledge between social contexts, and the use of that knowledge to improve practice. We will examine the processes themselves, and the role of human agents within those processes.

### Research questions

1. KTA processes: a) What KTA processes are initiated through the CoPs? b) How well do the three dimensions (evidence, context, and facilitation) proposed in the PARIHS framework describe the emergent patterns of knowledge flow? c) To what extent does KTA involve an interaction between explicit knowledge and tacit knowledge?

2. Roles of human agents: a) What roles are evident among those who participate in these processes? b) How does the active involvement of knowledge users in the KTA process influence knowledge utilization? c) What factors support or hinder effective involvement in KTA processes?

## Methods/design

### Conceptual framework

The conceptual framework that informs the study is the PARIHS framework [32-38]. As described earlier, PARIHS suggests that successful knowledge transfer depends on the interplay between three dimensions: the level and nature of the evidence being transferred, the nature of the organizational context where the evidence is being implemented, and the way in which the implementation process is facilitated. Kitson and colleagues suggest that knowledge transfer succeeds when evidence is coherent and relevant to the context where it is implemented, when local contexts have the capacity to adapt to useful new information, and when a process of enabling facilitation helps practice members to understand, absorb, and apply the new knowledge [34].

We will use PARIHS to inform the case studies, focusing on KTA processes in and through a CoP. We will observe and record the facilitative techniques used by the CoPs involved in the case studies; we will identify and catalogue the types of evidence assembled by the CoPs; we will note the prevalence of tacit and explicit knowledge within KTA processes; we will identify the characteristics of frontline contexts where the knowledge is directed; we will note the roles played by those who participate in these processes; and we will inquire among participants about the behaviour changes that result from these KTA processes.

### Overall implementation approach

This study will use a multiple case study design (nine cases over three years). Each case will yield a unique narrative, framed around the PARIHS dimensions; together, through cross-case analysis, the cases will shed light on how CoPs approach their knowledge exchange initiatives, and how they encounter challenges and succeed when bringing knowledge to action. Data will be collected using observation, semi-structured interviews, key informant interviews, and document analysis. Findings will be explored in annual stakeholder conferences, and in a final workshop involving participants and researchers from other Canadian knowledge networks.

Our case study design is appropriate for in-depth explorations of complex social phenomena within their natural contexts [75-77]. Case study research is used to describe and explain complex social phenomena occurring within and across organizational boundaries, such as processes that occur within and through CoPs and that extend to frontline settings [78]. Multiple case study research is appropriate when researchers want to understand a complex social phenomenon that is enacted in diverse situations [79].

**Figure 1:**
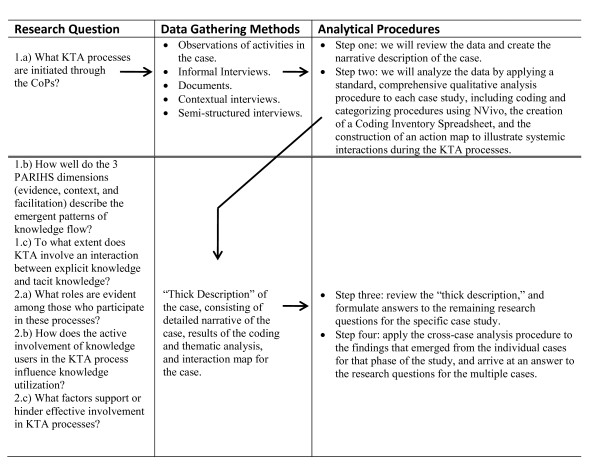
**Research questions and associated data-gathering and analytical methods**.

The project will be segmented into three twelve-month phases. Each phase includes three case studies, for a total of nine cases. One principal investigator (PI) will be responsible for one case in each phase. The nominated principal investigator (NPI) will be responsible for the cross-case analysis at the end of each phase. A total of nine cases is appropriate for a multiple case study design [79,80]. Each case will be subjected to an analytic process that generates an individual case report. The cases from phase one will be the basis for a cross-case analysis; the three cases from phase two, together with the phase one cases, will be used in a cross-case analysis at the end of phase two; and the cases from phase three, together with the analysis from previous phases, will be used in the cross-case analysis at the end of the project. Figure 1 shows the relationship between our research questions, data gathering methods, and analytical procedures.

## Phase one

### Sampling

In phase one, we will use purposive sampling to identify KTA initiatives within the CoPs. Each case will be conceived of as KTA processes mobilized around a specific body of evidence, that involve attempts to facilitate the adoption of new ideas or approaches within one or more frontline context. The case is not the CoP, but rather is the CoP's focus on a specific KTA objective. Each case will therefore consist of: a KTA objective established by the CoP leaders; activities undertaken to achieve that objective; the CoP members who carry out the activities and knowledge users who participate in the ensuing interactions; technologies that enable collaboration and communication; evidence that is amassed and/or translated to achieve the objective; and places where knowledge exchanges occur, and where knowledge users attempt to integrate the new knowledge into their practices.

### Data Collection

We will collect data through the following methods: observations of case study activities; informal interviews; semi-structured interviews; and identifying and obtaining copies of documents relevant to the case.

For each case, we will observe: CoP planning meetings, CoP interactions with potential knowledge users, CoP interactions with SHRTN planners/managers, and knowledge user interactions in their practice settings as they integrate the knowledge into the practice. The researcher will observe interactions among participants and will create a detailed record of the interactions that take place. To help control for observer bias, this record will be descriptive, making no reference to the conceptual framework or any other theories or models. Later, when recording the field notes, the researcher will make notes on possible patterns that are emerging, and will then explicitly consider how the case illustrates (and conflicts with) the interplay of PARIHS dimensions in the KTA process, and whether an interplay of tacit and explicit knowledge is evident.

The researcher will also note the ways in which knowledge users are involved in the KTA processes. Observers will use the involvement levels suggested by Stauffacher and colleagues [81], and will note instances when knowledge users passively receive information, are consulted for input, are asked to collaborate with knowledge providers, and are empowered to act with the knowledge provided. The researcher will note the roles played by participants in the case by using an observational tool derived from research on task and maintenance roles in small groups [82-87]. The tool is essentially a grid that allows an observer to record task-related behaviours (including such things as defining a problem, offering an opinion, providing information), maintenance behaviours (including harmonizing relationships, supporting teammates), and individualistic behaviours (including blocking, digressing) of group members, and that allows for the identification of recurring interaction patterns.

The researcher will also note how stakeholders attempt to use knowledge that is accessed through the case's KTA processes. Knowledge use will be conceived of as instrumental, conceptual, and symbolic [12,18,88-90].

Observations will be made using ethnographic methods to create a narrative description of what happened in each case [91,92]. Observers will be trained in advance, and will share their notes to ensure consistency. The precise logistics of observations will vary with the activities that occur in each case, but our discussions with stakeholders have led us to anticipate that in each phase we will observe 24 virtual sessions, three face-to-face meetings, and 15 on-site knowledge-user interactions.

Field notes will be written on the day when observations are made, using a structured format derived from the ethnographic literature [91-93]. Entries will begin with a description of what was observed and heard, followed by a section with personal impressions, emerging interpretations, and concerns. Entries will conclude with reflections on the research design and recommendations for changes to the approach.

When needed, we will conduct informal interviews to inquire into the meaning of the situations that we observe. These interviews will allow us to describe accurately the participant's experiences. The interview transcript will be shared with the interviewee, who will have the opportunity to correct errors and add information. We anticipate the need to conduct six informal interviews for each case, or 18 in each phase.

We will conduct formal, semi-structured interviews with CoP leaders at the start of the case, to help us understand the key features of the case from the perspective of the CoP team. See Additional File 1 for the draft interview protocol for knowledge users. We will want to hear about the CoP objective, who is involved, what activities will occur, when they will occur, what knowledge or evidence is being assembled, where it is sourced, what organizational contexts might receive the knowledge, and what facilitative mechanisms will be used. These data will help us plan the logistics for data collection, and will create a baseline to use later when we consider the success or shortcomings of this particular KTA process. The interview transcript will be shared with the interviewee, who will have the opportunity to correct errors and add information. We expect to conduct from one to three preliminary semi-structured interviews for each case, for a total of nine in each phase.

During these preliminary interviews and observations, we will ask to be provided with any documents that the CoP is using to inform the KTA exercise. These documents will be reviewed as they are gathered, and will be stored in a central location pending the analytical procedures. The documents will be considered examples of explicit knowledge relevant for the case.

As the case draws to a close, we will conduct semi-structured interviews with CoP leaders and knowledge users. These interviews will be structured in terms of the PARIHS dimensions and will include questions to help us understand the interplay of explicit and tacit knowledge in the case. The interviews will allow participants to look back on the experience, and reflect on the successes and challenges that they encountered. For each case, we expect to interview up to two CoP leaders, one KB, and five knowledge users, for a total of 24 in each phase.

Data gathering will conclude when saturation is reached. Each method is designed to produce data needed for the analytical procedures that we are using to answer each research question.

### Data analysis

Our analytical strategy is based on Wolcott's notion of the analytical objectives of qualitative inquiry: to describe the activities, people, places, and things involved in the case studies; to analyze how the KTA process unfolds by revealing systematic interactions; and to interpret these descriptions and analyses to arrive at a sense of what it means [94]. Our approach seeks to understand the unique features of each case and the social phenomenon represented across all cases [79].

In each phase, the analysis has four steps. In step one, we will review the data and create the narrative description of the case. In step two, we will analyze the data by applying a comprehensive analytic procedure. In step three, we will review the narrative description and the results of the analysis, and formulate answers to our research questions. For case studies in each phase, a single researcher will be responsible for carrying out the first three steps. In step four, the NPI will perform a cross-case analysis of the findings that emerged from individual cases.

In the first analytical step, one researcher will read through the data from beginning to end, making notations and memos and reflecting on the research questions. During this review, the researcher creates the narrative description of the case. In creating the narrative, we will write a 'thick description' [95] of events in each case, including descriptive commentary on the following: the knowledge that is the basis for the case study; the potential recipients of the knowledge; facilitative mechanisms used to help knowledge users understand, adapt, and use the knowledge in their practices, and the integration of the new knowledge into practice; the involvement of knowledge users in the KTA processes; and the emergence of leaders, and the characteristic forms of leadership. To ensure that the case studies can be compared in step four, the research team will agree on a table of contents for each case study. The researchers will meet via teleconference every two weeks during the analytical process, to ensure that their work remains aligned.

When a descriptive account for a case has been completed, the draft will be circulated among the researchers at the other research sites, and will also be provided to three key informants. Suggestions for revisions will be returned to the author of the account, and the final draft will be written. The draft will be considered complete when the researchers agree that it provides a coherent and comprehensive account of the case that sheds light on the research questions.

The second analytical step involves the comprehensive analysis of the data using coding and categorizing procedures [92,93,96,97]. We will not use a set of predetermined categories to guide this process, but rather will use a technique that allows codes and themes to emerge from a thorough review of the data. Given the amount of data we will accumulate, we will use NVivo for this step. The researcher will begin by reading through the full dataset a second time, using NVivo to make notations and create codes. At the end of this step, the researcher will create a code book consisting of a numeric identifier for each code, the code name and description, cross references to the code's location in the data set, and the number of data sources where the code originated. The researcher will then review the data a third time, locating instances of specific codes that were previously missed. This will be helpful for codes that had emerged late in the coding process.

The codes will be combined into a coding inventory spreadsheet to help us understand the relative importance of specific codes in the dataset. This exercise will allow us to confirm that codes are firmly grounded in the data.

We will then theme the data by working as a team with a clustering technique developed by the Institute for Cultural Affairs [98,99]. The technique will allow us to group all of the codes into thematic clusters, and then to assign a name and description to each cluster. The team will comment on and revise the descriptions and names until they agree that the wording reflects the meaning of the cluster. At the end of this step, we will develop a visual representation to depict KTA processes as systematic interactions among the thematic variables using the procedures recommended by Argyris for the creation of an interaction map to illustrate systemic learning patterns within a human system [100].

Step one provides the narrative account and step two provides the analytic account of the case. Together, these two analytical steps will answer research question 1a: What KTA processes are initiated through the CoPs?

Step three involves an interpretive process to answer the remaining research questions. The PI responsible for the case will, in effect, pose each question to the descriptive narratives, themes and interaction map produced in the previous steps. For example, the responsible PI will ask, What does the case tell us about how the three PARIHS dimensions describe emergent patterns of knowledge flow? The PI will review the narratives and write an answer to the question.

Together, the results of these three analytical steps constitute the thick description of each case. The description includes a detailed narrative account, a set of explanatory themes, an interaction map, and answers to each research question.

In step four, the NPI will review the three case reports from the phase to create a narrative description covering these topics: what the cases reveal about KTA processes mobilized through CoPs; developmental phases evident across the cases; people involved in the cases, and the roles they play; the results achieved in the cases; ways in which the cases differ, and what might account for the differences; and ways in which the cases are similar. Next, themes will be compared and themes that are evident across all cases or are unique to only some (or one) cases will be identified. To facilitate this, the NPI will create a table listing all themes identified in the cases, indicating whether the theme is of high, medium, low, or no importance to each case included in the analysis. The NPI will also consider if, upon looking across all cases, any additional themes are evident. New themes identified will be described in detail including a narrative description, brief description and proposed name. The NPI will also record how the theme is grounded in the various cases, giving three examples per case.

Next, the NPI will compare the interaction maps by noting salient points, documenting similarities evident across two or more maps, and noting unique features of specific maps. The NPI will consider whether a new map (or maps) could be created that abstracts features from specific cases to create a broader depiction of interactions in two or more cases, and if warranted, the NPI will create the new interaction map(s). Finally, a narrative account of the results of the analysis and the functioning of any new maps that have been created will be written.

Finally, the NPI will conduct a comparative analysis of the answers to the research questions. To start, the NPI will create a grid with the research questions in the left column, and summaries of the answers provided by each case in the remaining columns. The NPI will then compare the answers afforded by the cases to each question, noting differences and similarities. Where there are differences, the NPI will seek an explanation in the unique characteristics of the cases; where there are similarities, the NPI will consider whether they are sufficient to warrant the construction of a mid-level theory related to the question. A narrative description of how all of the cases combine will be written to answer each question. The description will highlight similarities and differences across the cases, and will offer suggestions to explain these similarities and differences. This step concludes with a review of the cross-case analysis by the research team. Based on comments and suggestions that are elicited from the team, a final draft will be prepared.

### Phases two and three

For phases two and three, we will again use purposive sampling to identify CoPs and informants engaged in KTA initiatives, and will use the selection criteria described earlier. In addition, cases will be selected using replication logic [101]. This methodological feature ensures a focus on cases to confirm or challenge and refine emerging findings. We will select one case that resembles and two that differ from the cases in the previous phase. A case will be considered different if the knowledge being mobilized or the mobilization process is different (*e.g*., if phase one focuses on 'push' strategies to implement knowledge, then in phase two we will identify more cases involving 'pull' strategies). A case will also be considered different if the organizations that are expected to accept and use the knowledge are different from those in phase one (*e.g*., if during phase one the cases primarily concerned long-term care homes, then during phase two we will identify cases focusing on community care agencies). Finally, a case will be considered different if the facilitation methods used to move the knowledge into practice are different (*e.g*., if phase one cases all used educational sessions as facilitative mechanisms, then in phase two we will attempt to identify cases involving the formation of collaborative teams, or joint planning and problem-solving sessions).

Phases two and three will use the same data collection methods as phase one. Additionally, they will use the same analytical procedures as phase one, with one difference. During the phase two and three cross-case analysis, findings from the previous phase(s) will be added after the comparison of the current cases is complete.

### Ethics approval

This protocol received approval from the University Human Research Ethics Committee of Concordia University on November 2, 2010 (reference number UH2010-115).

## Discussion

### Engaging the stakeholder community

Our research focuses on KTA processes in a network intended to mobilize knowledge in service of clinical care and policy formation, and we believe it is essential that our findings be useful for stakeholders and others interested in these issues. In keeping with best practices in planned change in human systems [102-104], we conceive of the project itself (and not just its results) as a potential instrument of change. Project activities are designed to engage stakeholders, solicit feedback about the project, and disseminate findings. To this end, we will again use the varying levels of stakeholder involvement in research suggested by Stauffacher and colleagues [81].

We will hold quarterly meetings with our KTA advisory team. At the end of each phase, we will convene a stakeholder conference to present findings to members of the SHRTN collaborative. The conference will be a collective sensemaking forum, where results are presented and small groups suggest interpretations for the researchers to consider, and also how the findings might be used to improve network performance. Forums of this sort have been an effective means by which broad stakeholder groups can create common ground for collective action [103].

At the end of phase three, we will host a KTA network summit where we will share results with others who are conducting research in knowledge exchange networks. The guest list for the summit will depend on what groups are active at that time. Participants in the summit will present their findings, and discuss research gaps and strategies for improving our ability to move relevant knowledge into frontline contexts.

### Assuring the quality of our findings

To assure the trustworthiness of our data, we draw on Patton's suggestion that each researcher have the qualifications to carry out the study [105]. Our team includes skilled researchers with a combination of formal training and practical experience in the use of all methods in this study. Our project design includes methodological training for all research associates who participate in the data gathering and analysis.

Lincoln and Guba state that qualitative research must produce credible, transferable, dependable, and confirmable results [93]. The credibility of our findings will be tested through member checking, and through quarterly and annual sensemaking sessions with stakeholders. Transferability will be assured through a 'thick description' allowing readers to assess the applicability of the results to other contexts. Dependability will derive from the finding's internal coherence, which will be created through member checking, reviewing, and editing steps involving the full research team. Confirmability (which requires that conclusions be well grounded in data) will be assured through the coding and theming procedures of our analytical process. Numerous qualitative researchers have noted that triangulation of informants, situations, researchers, methods, and investigators helps to assure the trustworthiness of the results of a qualitative inquiry [106-108]. We provide triangulation in terms of informants, situations, researchers, data-gathering methods, and investigators.

Creswell and Miller suggest that validity in case study research depends on accurately representing the way in which participants view the phenomenon being studied, and the extent to which participants see the findings as credible [106]. We will use eight of the nine validity procedures they suggest: triangulation, member checking, disconfirming evidence, prolonged engagement, thick description, researcher reflexivity, collaboration, and peer debriefing.

### Importance of the research

This research will contribute to our understanding of the role and impact of CoPs in the KTA process, the developmental processes of CoPs, the importance of stakeholder engagement in KTA, and the use of PARIHS to understand these processes. We will generate new knowledge about the defining characteristics of CoPs operating in the health system, on leadership roles in CoPs, and on the nature of interaction processes, relationships, and knowledge exchange mechanisms. Our work will yield a better understanding of the factors that contribute to the success or failure of KTA initiatives. We have designed the project to be consistent with the suggestion by the PARIHS group for framing KTA research in a collaborative (including researchers, practitioners, and others) to assess the usefulness of PARIHS for revealing the interdependent nature of KTA processes that can lead to the design of interventions to improve the uptake of relevant knowledge.

This research will improve our understanding of how local caregiving contexts interact with KTA programs. As called for by Greenhalgh and colleagues, we will produce detailed reports of the unique features of the local contexts being studied [39]. Moreover, our participatory designs will allow stakeholders to influence the practical usefulness of our findings. Thus, our project will also contribute to improved health services delivery for seniors.

From their participation in this project, it is clear that the SHRTN collaborative's stakeholder community believes in the importance of this research. We will hold quarterly meetings with an advisory group and annual stakeholder conferences where we will discuss the research findings to empower stakeholders to build capacity for evidence-based action. We are also linking with others who are studying KTA processes with the PARIHS framework to fertilize each other's efforts and spawn additional research collaborations that build on our collective results.

We anticipate that the methods developed through this project will be adaptable to other contexts. We believe that this proposal is the first multiple case study research project focused on KTA processes in Canada. The approach combines a stringent focus on the details of specific instances of KTA, along with a structured process to aggregate the individual results and arrive at more transferable lessons.

## Competing interests

The authors declare that they have no competing interests.

## Authors' contributions

JC conceived of the study and developed the original protocol, and wrote the first draft. PS and AK made important, substantive contributions to the protocol, and reviewed and commented on multiple drafts. All authors read and approved the final manuscript.

## Supplementary Material

Additional file 1 **Draft interview protocol for knowledge users**. Click here for file
