# Supplementary material for: Knowledge-to-action processes in SHRTN collaborative communities of practice: A study protocol
Source: Implement Sci. 2011 Feb 11;6:12. doi: 10.1186/1748-5908-6-12 (PMC3055177; doi:10.1186/1748-5908-6-12)
Supplement: Additional file 1 — Draft interview protocol for knowledge users. [file 1748-5908-6-12-S1.PDF]

Additional File 1 - Draft Semi-Structured Interview Protocol

Note: This version of the protocol is tailored to knowledge users. The questions would be adjusted for interviews with CoP leaders and other stakeholders.

General Questions about the Case

1. Can you tell me about your current job and role, and how you came to be interested in this <descriptor for the case>?

2. What were you trying to achieve by participating in <descriptor for the case>?

Evidence Questions

3. To meet this objective, what type of knowledge (e.g. research evidence, best practices, procedural information, etc.) is most useful? (Probe: What type of knowledge ended up being made available through the case? Did you find this knowledge to be trustworthy?)

4. Was the knowledge that you accessed through <descriptor for the case> relevant for your situation?

5. Is this knowledge the only basis on which you would make a decision, or would other factors also be involved in a decision?

6. Was it clear to you how you could use this knowledge to achieve your objective? Did you need to take steps to make the knowledge useful for your practice?

7. Did the knowledge you accessed through <descriptor for the case> make sense based on your own work experience?

8. Did you also ask your [patients/residents/clients] about their perspective on these issues? Do you consider their views and experiences when you make your decisions?

9. What was most useful for you about participating in <descriptor for the case>? [Probe: was it the knowledge that people gave to you, or what you learned through conversations, or the new contacts and relationships you made, or something else?]

Context Questions

10. In your workplace, will it be difficult or easy to introduce changes in how things are done, based on the knowledge you accessed through <descriptor for the case>?

11. Do you have the people, equipment and money needed to make the changes?

12. Are the changes consistent with your organization's strategic plan or values?

13. Will your colleagues and superiors be receptive to the change?

14. What will your role be in implementing the change? Can you identify any other roles among your peers and others?

15. How will you know if the change has succeeded? How do you evaluate performance in your organization?

16. When an improvement or change is introduced into your workplace, are lots of different people given a chance to design and implement the change, or is it left in the hands of just a few people?

17. Would you say that there is a great deal of open communication and dialogue in your workplace?

18. Would you say that things are continuously improving in your workplace?

19. Are you and your colleagues treated as individuals in your workplace?

20. Are your <patients/residents/clients> treated as individuals by your organization?

Facilitation Questions

21. While you have worked on this change, did your superiors usually tell you what to do, or did they usually ask you what you thought needed to be done?

22. What were the most important activities in this entire process, that either helped to make the change happen, or that blocked the change? Are these kinds of activities typical of what happens in this organization?

23. What did you learn through this process? What new skills did you develop?

24. Has participating in this process changed the way you will do your work in the future?
